# Open Tubular Lab-On-Column/Mass Spectrometry for Targeted Proteomics of Nanogram Sample Amounts

**DOI:** 10.1371/journal.pone.0106881

**Published:** 2014-09-15

**Authors:** Hanne Kolsrud Hustoft, Tore Vehus, Ole Kristian Brandtzaeg, Stefan Krauss, Tyge Greibrokk, Steven Ray Wilson, Elsa Lundanes

**Affiliations:** 1 Department of Chemistry, University of Oslo, Oslo, Norway; 2 Unit for Cell Signaling, Cancer Stem Cell Innovation Center, Oslo University Hospital, Oslo, Norway; Swiss Institute of Bioinformatics, Switzerland

## Abstract

A novel open tubular nanoproteomic platform featuring accelerated on-line protein digestion and high-resolution nano liquid chromatography mass spectrometry (LC-MS) has been developed. The platform features very narrow open tubular columns, and is hence particularly suited for limited sample amounts. For enzymatic digestion of proteins, samples are passed through a 20 µm inner diameter (ID) trypsin + endoproteinase Lys-C immobilized open tubular enzyme reactor (OTER). Resulting peptides are subsequently trapped on a monolithic pre-column and transferred on-line to a 10 µm ID porous layer open tubular (PLOT) liquid chromatography LC separation column. Wnt/ß-catenein signaling pathway (Wnt-pathway) proteins of potentially diagnostic value were digested+detected in targeted-MS/MS mode in small cell samples and tumor tissues within 120 minutes. For example, a potential biomarker Axin1 was identifiable in just 10 ng of sample (protein extract of ∼1,000 HCT15 colon cancer cells). In comprehensive mode, the current OTER-PLOT set-up could be used to identify approximately 1500 proteins in HCT15 cells using a relatively short digestion+detection cycle (240 minutes), outperforming previously reported on-line digestion/separation systems. The platform is fully automated utilizing common commercial instrumentation and parts, while the reactor and columns are simple to produce and have low carry-over. These initial results point to automated solutions for fast and very sensitive MS based proteomics, especially for samples of limited size.

## Introduction

As cancer medicine evolves towards personalized treatment, the need for high throughput, selective and sensitive methods for monitoring key diagnostic-value biomolecules is increasing [1]–[3]. In this regard, much diagnosis-relevant sample measurements are expected to be performed at the gene/mRNA level, and several high-throughput genetic testing methods have been developed for e.g. breast, colon, prostate and colorectal cancer [4]–[7]. Still, changes in the gene expression might not be reflected on the level of protein expression or function [8], [9]. Measuring proteins is therefore important for understanding the link between gene, gene variants and their contribution to the pathology of disease [10]. Although protein identification/measurement with e.g. Western Blotting (WB) has been predicted to have an increasingly larger role in clinics, WB has several disadvantages, e.g. limited numbers of analytes per test and selectivity issues [11].

MS based approaches can provide additional information with higher throughput, selectivity or sensitivity compared to e.g. WB, ELISA, DNA and mRNA sequencing [11]–[14]. However, standard protein MS methodologies can be very slow; cleaving proteins into MS-friendly peptides can demand overnight treatment (typically performed with the enzymes trypsin and/or Lys-C), and comprehensive LC-electrospray ionization (ESI) MS processes can take tens of hours [15]–[24]. The lengthy LC-MS processing is much related to the inability of standard LC to sufficiently separate all of the peptides in a mixture (which can be hundreds of thousands), that in turn compromises the ESI step (i.e. co-eluting peptides suppressing the signal of one another). Therefore, a goal in the LC-MS based proteomics is to develop novel LC columns/materials that provide improved separation, i.e. high peak capacity; contemporary approaches include development of monolithic materials (silica and organic [25], [26]), ultra high performance LC (UHPLC) [27] and fused core particle materials [28].

The rate-limiting step of the enzymatic cleaving of proteins is the use of relatively slow in-solution procedures. Thus, another goal is to develop procedures that accelerate the enzyme reaction step: one approach is the use of immobilized enzyme reactors (IMERs), as they have several advantages compared to standard in-solution/in-gel approaches, such as larger enzyme to substrate ratio, higher digestion efficiency, long-term stability and reusability [29], [30].

For improving both LC separation and accelerate enzymatic digestion, we have recently demonstrated the proof of principle of using a (manually operated) “lab-on-a column” system with open tubular columns (instead of particle packed/polymer-filled columns used nearly invariably in today's LC laboratories). Specifically, trypsin was immobilized on to 20 µm ID polymer-wall coated capillaries (OTERs), cleaving an isolated small cell lung cancer (SCLC) marker (ProGRP) into peptides that were subsequently separated by high peak capacity PLOT LC columns (10 µm ID [31]), with cleavage and LC-MS run time of totally 40 minutes.

Although known to be a path to ultra-high resolution and sensitivity, PLOT columns have hardly been used in LC-MS (notable exceptions are described in references [31]–[36]) much due to incompatibilities with commercial LC instrumentation and detectors. These issues are to a large degree resolved [31], [35]. In addition to high LC performance, PLOT columns are simple to manufacture, with a high degree of reproducibility [34], [36].

We here describe a further developed OTER-PLOT system that enables fast enzyme cleavage and selective detection of target proteins in biological samples. The OTERs have been improved by e.g. increasing reactor length (volume) and immobilizing with a combination of trypsin and Lys-C, and the system is now fully automated, using commercial LC-MS instrumentation and software. By utilizing the narrow bore 10 µm ID PLOT columns, high sensitivity is achieved, which is important for detecting proteins present at low concentrations in samples of limited availability. The system performance is demonstrated by detecting targeted Wnt-pathway proteins from cell lines and xenograft tissues.

## Materials and Methods

### Chemicals, reagents and materials

All chemicals, reagents and materials were obtained from commercial sources and chemicals/reagents were of analytical grade. See Chemicals, reagents and materials S1 in File S1, for more details.

### OTER preparation

For a visual description of the production of the OTERs, see Video S1. Figure S1 in File S1 illustrates the reaction chemistry of the OTER. In short; the wall-polymerization solution consisting of 0.08 g HEMA, 0.02 g VDM, 0.60 g 1-heptanol and 0.0001 g AIBN, was filled into a 20 µm ID pre-treated fused silica capillary using an in-house made pressure bomb, and the capillary ends were sealed by sticking the ends into septa (originally intended for use in gas chromatography (GC) injection systems). The polymerization was performed in an oven (originally intended for GC column heating) at 65°C for 5 hours, followed by 80°C for 5 hours. The polymerized capillary was dried with nitrogen for 30 min. Immobilization was performed by flushing the column with the appropriate enzyme solution (trypsin: 2.5 mg/mL trypsin and 0.25 mg/mL benzamidine, Lys-C; 5–15 µg/mL Lys-C and 0.5–1.5 µg/mL benzamidine, Trypsin/Lys-C mix; 20 µg/mLTrypsin/Lys-C and 0.2 µg/mL benzamidine, solved in 20 mM phosphate buffer, pH 7.4) for 3 hours. The OTER was subsequently filled with 50 mM ammonium acetate, pH 6–7 and stored at 4°C.

For detailed description of production of the pre-columns and polystyrene divinylbenzene (PS-DVB) PLOT LC columns (previously described in [37]) see Methods S1 in File S1.

### OTER-PLOT system set-up

**Figure 1:**
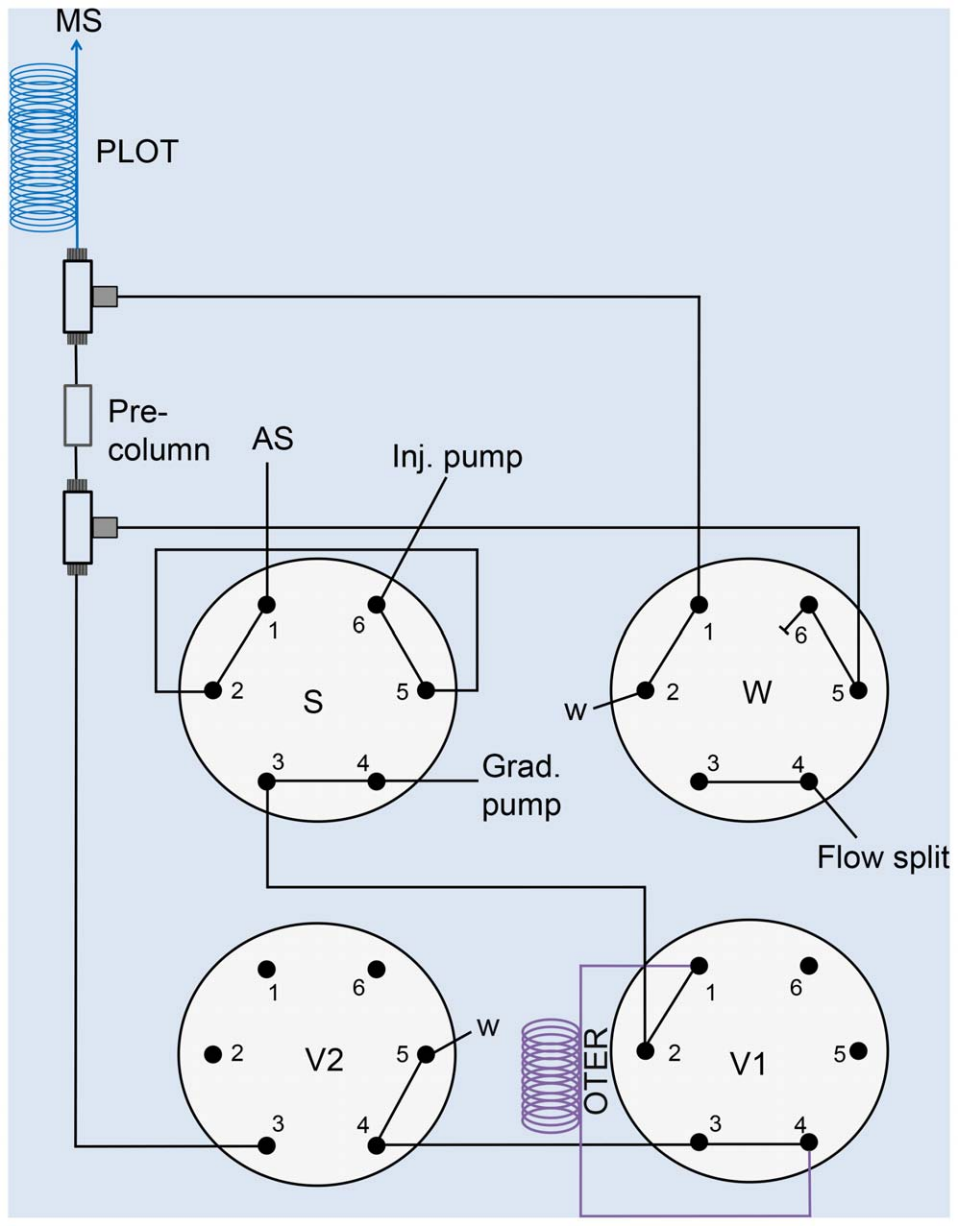
System set-up using an Easy-nLC1000 pump equipped with two extra standard 6-port valves (V1 and V2). See Animation S1 for more detailed valve position during the platform performance. The OTER is kept in a column oven at 37°C.

The manual OTER-PLOT system is described in detail elsewhere [37] and in Methods S2 File S1. For the automated set-up (see Figure 1 and Animation S1) an Easy nLC1000 (Thermo Fisher Scientific, Bremen, Germany) pump was used for injection, column equilibration and gradient elution. Mobile phase A (MP A) contained 4% ACN in 0.1% FA, whereas MP B contained 0.1% FA in ACN. To incorporate the enzymatic reactor to the Easy nLC1000 pump, two automatic port switching valves were added; one Rheodyne 6-port valve (V1, Figure 1, IDEX Health & Science LLC, Rohnert Park, CA, USA) and one 10-port valve (V2, shown as a 6-port in Figure 1, VICI, Valco Instruments Co. Inc., Houston, TX, USA), both connected to the MS contact closure outputs and controlled by the MS instrument software. Note that valve V2 may be replaced with a 6-port valve and is illustrated as such for simplicity. Fused silica tubing (20 µm ID) was used between valves S, V1 and V2.

Five µL sample were loaded onto the injection loop, and an appropriate volume was loaded onto the OTER (4 meter (∼1.2 µL) connected using PicoClear unions (New Objective, Woburn, MA, USA)) at a flow rate of 0.5 µL/min (V1 with connecting ports 1 and 2 and V2 connecting ports 1 and 6). The OTER was conveniently placed inside a column oven (Mistral, Spark Holland) at 37°C. After loading, V1 was switched; connecting ports 1 and 6 and the tubing was flushed for 5 minutes with 100% MP A while the autosampler loop was flushed with 4% ACN in 50 mM ammonium acetate. V2 was switched, connecting ports 1 and 6 and the pre-column and PLOT column were equilibrated for 55 minutes at 40 nL/min. 2 µL 4% ACN in 50 mM ammonium acetate eluted the generated peptides from the OTER and onto the 50 µm ID x 4 cm butyl-methacrylate (BuMa) monolithic pre-column, with valve W connecting ports 1 and 2, for 4 minutes at 0.5 µL/min. The pre-column was used to trap and desalt the peptides. The flow was split to 40 nL/min by switching valve W to connect ports 1 and 6, and peptides were separated using a linear gradient from 0–40% MP B in 40 minutes, with a subsequent linear step up to 95% MP B in 10 minutes and kept at 95% MP B for 10 minutes, on a 10 µm ID x 500 cm long PS-DVB-PLOT column.

For analysis of pre-digested samples, valves V1 and V2 were set in the position connecting ports 1 and 6, and ports 1 and 2, respectively, and 1 µL sample was loaded onto the pre-column for 4 minutes at a flow-rate of 0.5 µL/min, with subsequent separation of peptides on the PS-DVB PLOT column.

For the comprehensive set-up, gradient elution was performed from 0–40% MP B in 250 minutes, with a linear increase to 95% MP B in 10 minutes and wash at 95% MP B for 20 minutes. For targeted investigations, linear gradient elution was performed for 40 minutes from 0–40% MP B, with a linear increase for 10 minutes to 95% MP B and a hold at 95% MP B for 10 minutes. The PLOT column was connected to a Q-Exactive Mass Spectrometer (Thermo Fisher Scientific) equipped with a 10 µm ID to 5±1 µm nanospray ESI-emitter (PicoTip, New Objective), with an applied voltage of 1.3 kV and capillary temperature of 250°C. The system could just as easily be connected to other mass spectrometers. e.g. a triple quadrupole MS for quantitative multiple reaction monitoring (MRM).

### Mass spectrometry

For data-dependent acquisition the full MS scan range was set to *m/z* 350–1850 and the 12 most intense ions were selected for fragmentation. The full MS resolution was 70,000 with an automatic gain control (AGC) target value of 1e6 and maximum fill time of 120 ms. The MS/MS resolution was set to 35,000 with AGC target value of 1e5 and maximum fill time of 120 ms. The ions were fragmented with a normalized collision energy (NCE) of 25, isolation width of *m/z* 2.0 and only ions with charge +2 - +6 were selected for fragmentation. The dynamic exclusion was set to 30 seconds. The Easy nLC1000 and Q-Exactive Orbitrap were controlled by Xcalibur software (v2.2, Thermo Fisher Scientific).

For preparation of the standard solutions, HCT15 cell lysate, tumor tissue and database search, see Methods S3 and S4 in File S1. A list of identified proteins can be found in Table S1. Other raw files and spreadsheets are available at http://www.mn.uio.no/kjemi/supplementary-data/bach/Open-tubular-lab-on-column-mass-spectrometry-for-targe/. In the unexpected event of web relocation of the raw data files, we will provide the new web address in the comments section of the online version of this paper.

### Ethics statement

The tumor tissue samples were from 6 week old female CB17 SCID mice xenografted with HCT15 cells that were treated with tankyrase inhibitor G007-LK, which was administered at 10 mg/kg daily (study described in Lau et al [38]). All animal experiments were approved by the Norwegian Animal Research Authority (NARA) and were carried out following accepted ethical standards. The experiments/procedures have thus been conducted in accordance with the laws and regulations controlling experiments/procedures of live animals in Norway, i.e. the Animal Welfare Act of December 20th 1974, No 73, chapter VI sections 20–22 and the Regulation on Animal Experimentation of January 15th 1996. In addition, Norway has signed and ratified The European Convention for the protection of Vertebrate Animals used for Experimental and other Scientific Purposes of March 18th 1986. The Norwegian legislation conforms in all respects with the basic requirements of this Convention and guidelines prepared in pursuance of it. All efforts were made to minimize the number and suffering of animals.

For details regarding the preparation of these samples, see Methods S3.4 in File S1.

## Results and Discussion

In order to further develop the prototype OTER-PLOT-LC-MS [37] into an applicable platform, several improvements were needed regarding the OTER and automation of the platform.

### OTER features and characteristics

**Figure 2:**
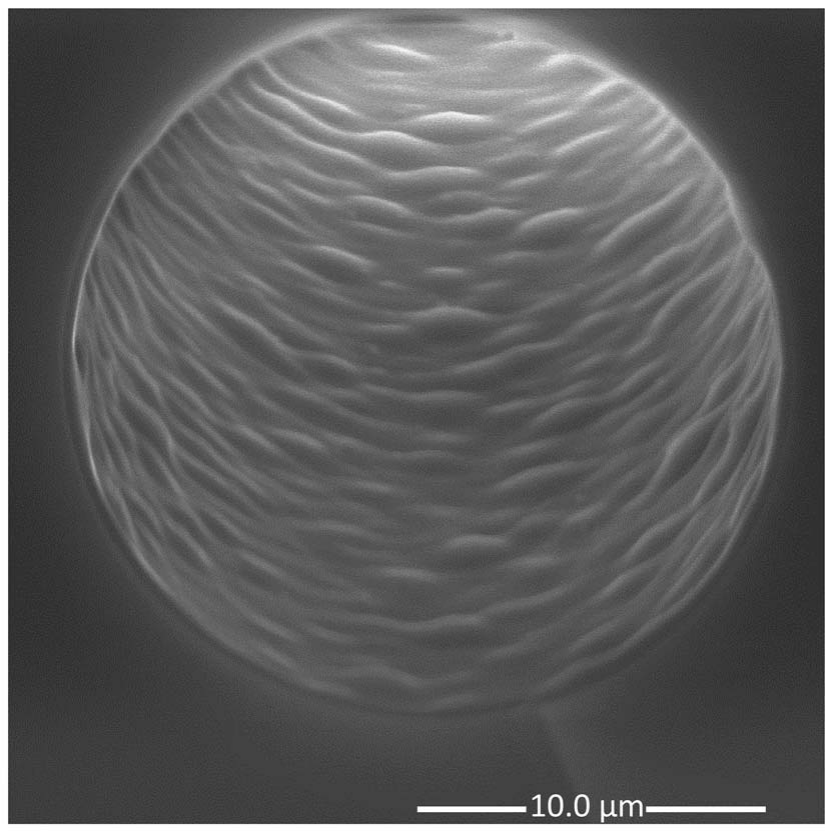
SEM image of the OTER (20 µm ID) (the immobilized enzymes are too small to be visualized in the image). The polymeric layer was ∼0.4 µm (dry).

In a narrow bore open format, open tubular enzyme reactors (OTERs) have been found to be easy to repeatedly prepare and with less backpressure (and thus short preparation times when filling reagents into capillaries with the pressure bomb) compared to monolithic and packed reactors [39]–[43]. To increase the effective surface area compared to previous efforts ([37]), longer reactors need to be used [44]. Increasing the reactor volume also allows for exploiting more of the available small sample. Changing the porogenic solvent in the polymerization solution from 1-decanol (used in [37] based on procedures described in reference [45]) to 1-heptanol, reactors of 1 meter (∼300 nL volume) were easily produced. A scanning electron microscope (SEM) image of the inside of an OTER can be seen in Figure 2; a thin polymeric layer of ∼0.4 µm (dry) with evenly spread globules make up the anchoring phase for the proteolytic enzymes.

**Figure 3:**
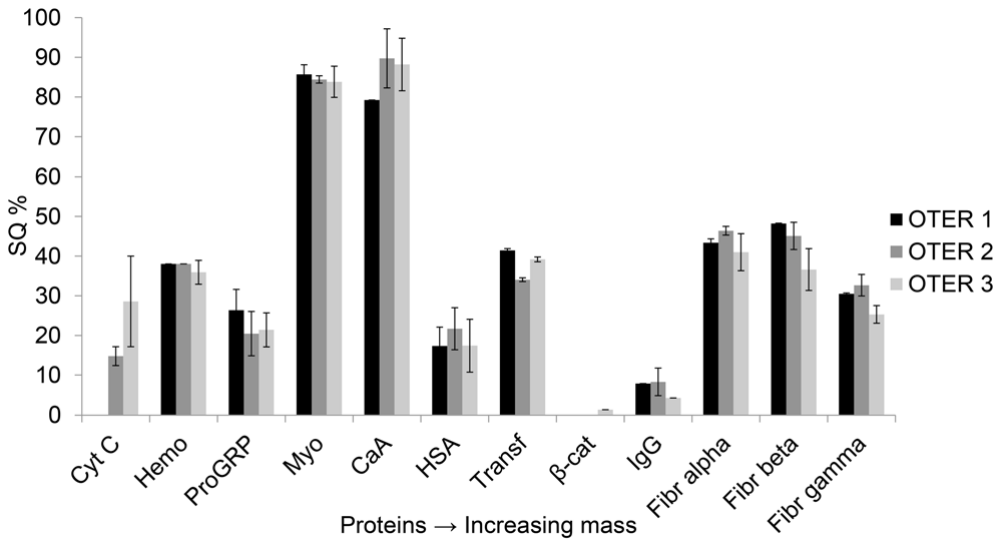
Repeatability of performance (defined as number of identified proteins and SQ%) of three individually produced OTERs. A 10 protein standard mix (equal molarity of each protein) was digested in the automated platform, OTER volume was ∼300 nL and three replicate injections were performed for each OTER.

Trypsin is the most used proteolytic enzyme for the conversion of proteins to peptides, also in micro scale IMERs. Only a few IMERs are reported immobilized with other enzymes such as Lys-C, pepsin, PNGase F, proteinase K and chymotrypsin [30], [44]. Choosing a combination of trypsin and Lys-C, which is often used in off-line digestion can provide a more complete digestion of proteins by reducing the number of missed cleavage sites, enabling improved repeatability and accuracy [46]. In initial investigations of reactor performance Lys-C, trypsin and Trypsin/Lys-C mix, were immobilized onto short 20 µm ID (∼60 nL) polymerized reactors and observed digestion of a standard protein, cytochrome C (commonly used to evaluate IMERs) was used for measuring reactor efficiency. The Trypsin/Lys-C reactor gave a 30% increase in cleaved protein, outperforming the Lys-C-only and trypsin-only immobilized reactors. With a reactor volume of ∼60 nL, only 2 out of 10 proteins in a standard mix were digested, but by increasing the length (and hence the volume) of the reactor 5-fold (∼300 nL), 10 out of the 10 proteins could be identified (Figure 3). A reactor digestion temperature of 50°C resulted in higher sequence coverage and more identified standard proteins compared to 22 and 37°C (Figure S2 in File S1). However, a reactor temperature of 50°C was associated with poor performance (less identified proteins) when longer (up to 2 h) digestion times were evaluated (Figure S3 in File S1), thus digestion at 37°C was chosen as it provided far more robust performance. As a compromise between time and throughput, 1 hour digestion time was chosen for the on-line analysis of the cell lysate and tumor xenograft samples. The reactors showed good repeatability of performance when three 1 meter (∼300 nL) individually prepared OTERs were used for digestion of the 10 protein standard mix (Figure 3).

The complementary combination of trypsin and Lys-C has, as already mentioned, been investigated in several off-line experiments, but to the authors' knowledge for the first time been exploited in the open tubular IMER format with a reactor volume that makes them very suitable for analysis of small samples.

### A fully automated nanoproteomic platform

**Figure 4:**
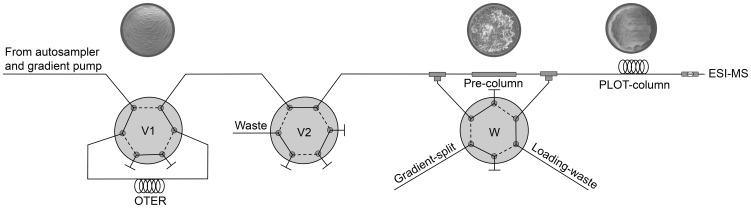
Graphical overview of the OTER-PLOT nanoproteomic platform.

Automated systems are usually required for high-throughput analysis. On-line protein digestion (with integrated OTER) and subsequent peptide separation (PLOT column) was performed with a fully automated platform using a commercial nLC system, with minimum dead volumes and low backpressures (<400 bar). A schematic overview of the platform is presented in Figure 4. Figure 1 (and Animation S1) provides a detailed description of the plumbing and valve positions of the platform.

Although other sensitive nanoflow columns in somewhat larger formats exists, e.g. 25 µm ID packed columns for peptide separations [47], PLOT LC columns are associated with good column-to-column production repeatability together with high performance and little carry-over [31], [34] and are well adapted to be coupled on-line to enzyme reactors [37].

**Figure 5:**
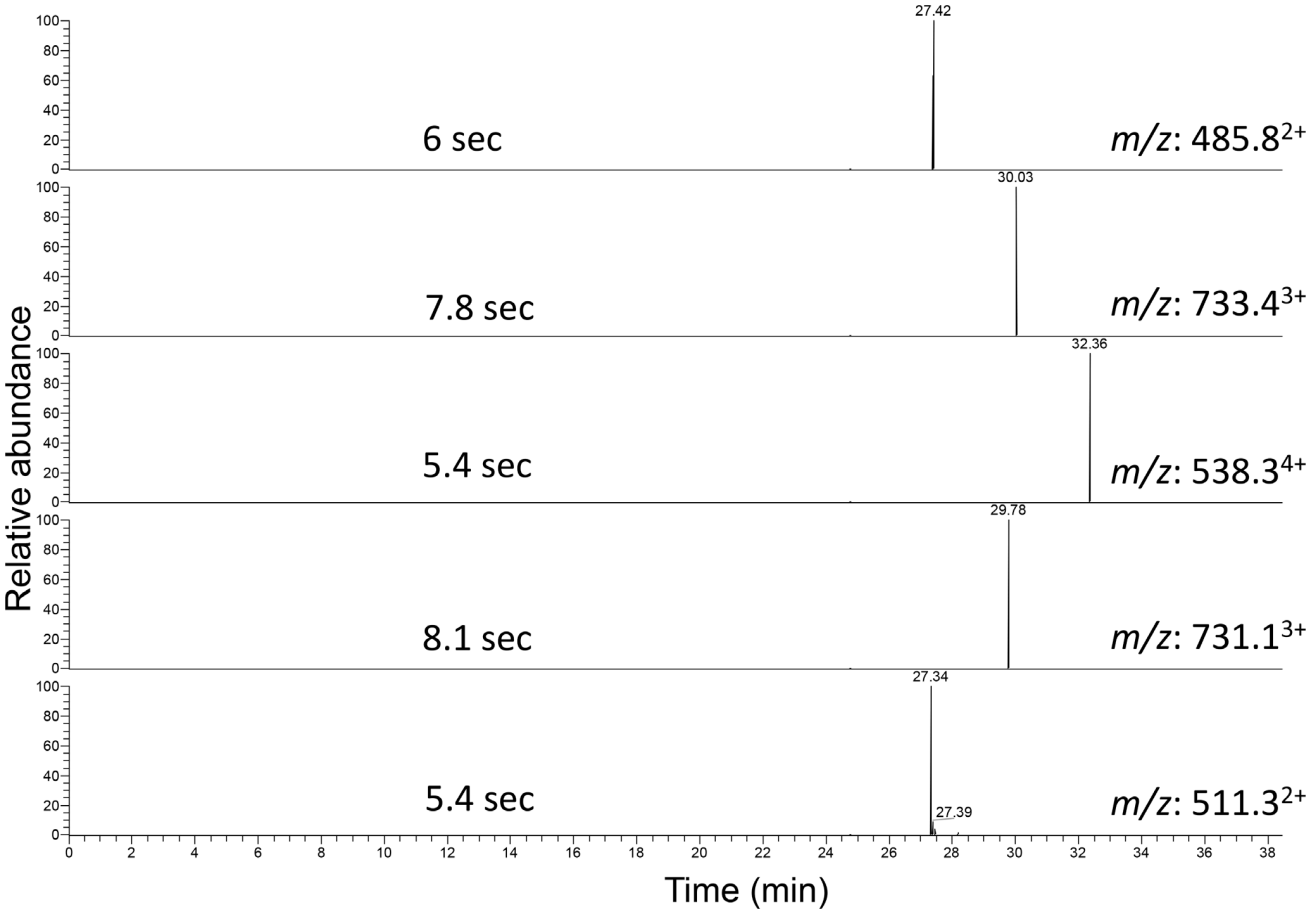
Extracted ion chromatograms of 5 chosen peptides from the 10 protein standard mix. The average peak width at 10% peak height (W_0.1_) was ∼6 seconds.

**Figure 6:**
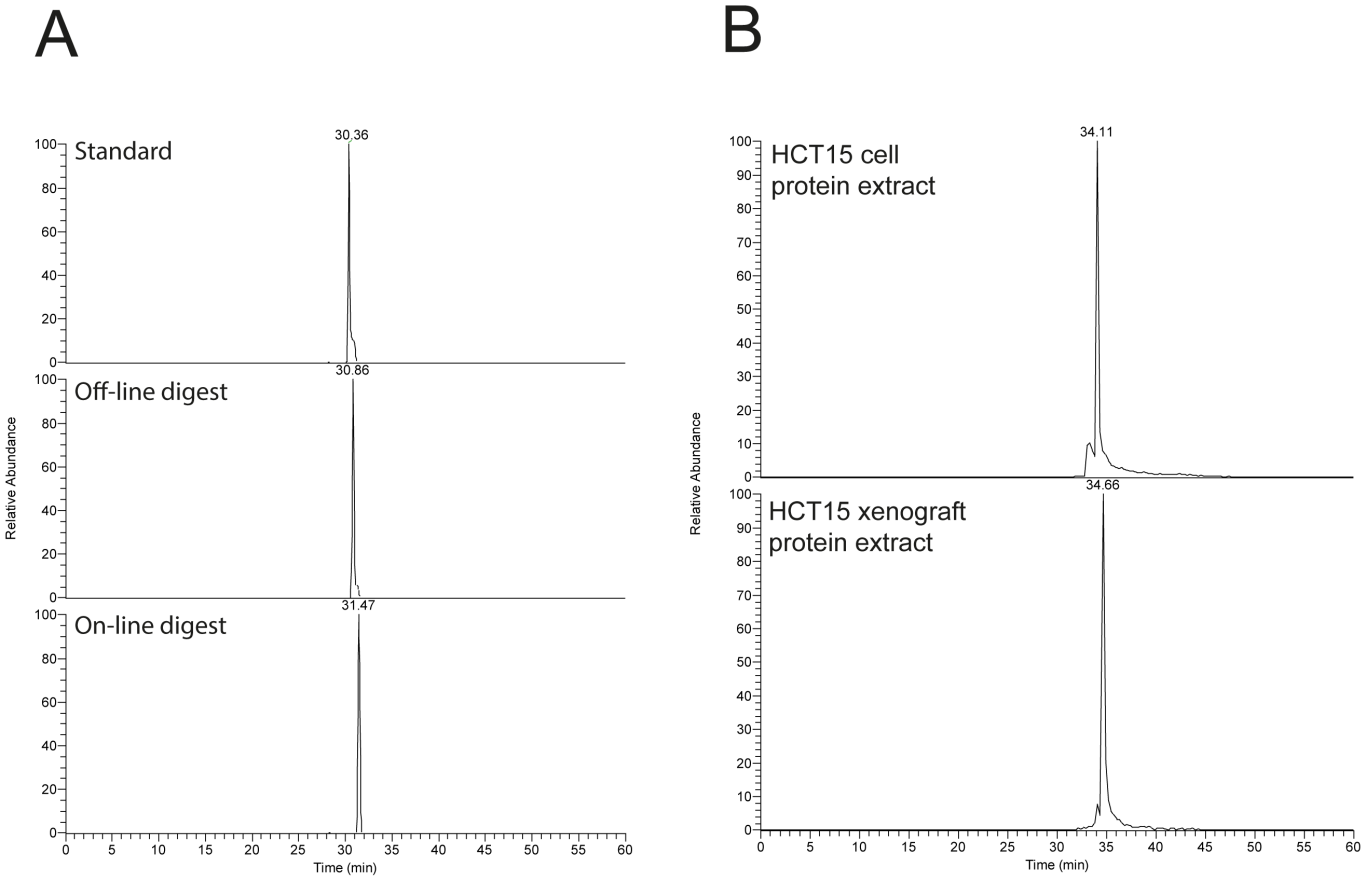
A. EIC of identified peptide TSVQPSHLFIQDPTMPPHPAPNPLTQLEEAR corresponding to Axin1 in standard mixture, HCT15 cell protein extract digested off-line and on-line. B. EIC of identified peptide HETGSHDAER corresponding to APC in protein extracts from HCT15 cell line and HCT15 xenograft, respectively. OTER volume was approximately 1.2 µL.

Figure 5 shows that chromatographic peaks can be very narrow using the automated OTER-PLOT platform (typical peak widths W_0.1_ are ∼6 seconds). Conveniently, the system set-up can be programmed to perform both conventional LC-MS (i.e. analysis of pre-digested samples) and OTER-PLOT performance, without any hardware changes. Figure 6 A and B show that peak shapes, retention times and peak widths obtained in traditional off-line digestion mode and OTER-PLOT mode are very similar (evaluated with signature peptides that were easily produced with both in-solution digestion and OTER), which is a testament to the robustness of the automated OTER-PLOT platform.

The OTER-PLOT run time including both protein digestion and LC-MS processing was typically 240 min (1 h digestion, 150 min LC run time) in comprehensive mode and 120 min (1 h digestion and 60 min LC run time) in targeted mode (which is our current main focus). However, these run times can be shorted and lengthened for faster analysis or higher peak capacities, respectively. All samples analysed in the platform were reduced and alkylated in advance, but as Kim *et al.* suggest this step might be avoided due to the enhanced rate of proteolysis that is provided by IMERs, that in many cases allows direct digestion of proteins without reduction and alkylation.

**Figure 7:**
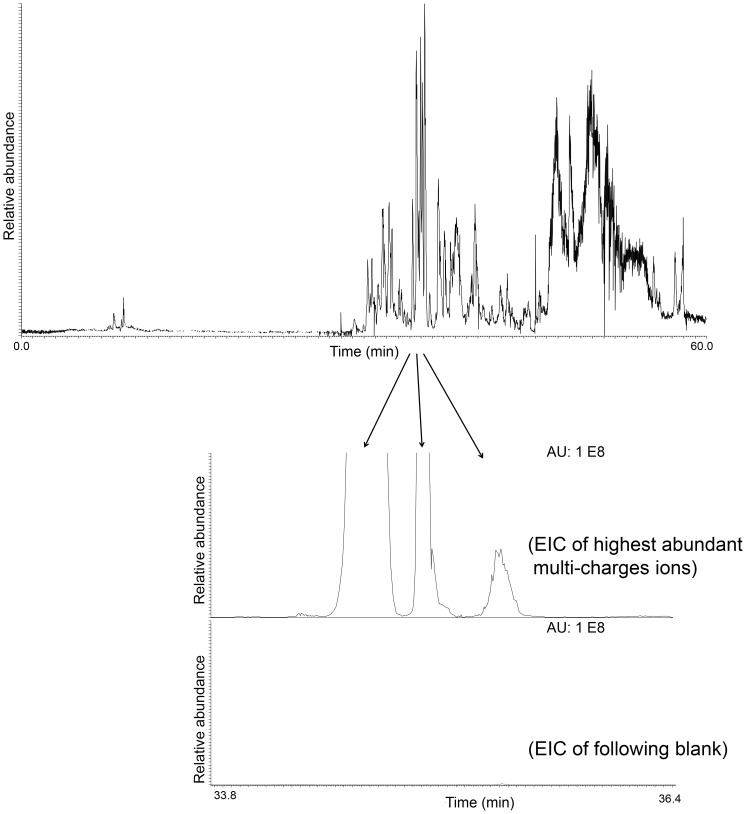
Carry-over of the highest abundant peptide ions in a blank following injection of a 10 protein standard mix. Upper chromatogram: TIC of the standard protein mixture, lower chromatograms; zoom-in of three of the highest abundant ions in the standard protein mix compared to the same ions in the blank (not able to extract). OTER volume was approximately 1.2 µL.

The platform carry-over was generally not detectable (Figure 7), but it should be mentioned that in rare cases up to 2.5% carry-over could be observed for certain peptides, when injecting very high concentrations of standard protein mix (300 µg/mL).

The platform, being automated and consisting of both integrated protein digestion and chromatographic separation in narrow open tubular columns is unique in its field today. Others have described related set-ups (using larger bore packed/monolithic columns), but none with e.g. a fully integrated IMER and simple commercial instrumentation. Kim *et al*. describe a semi-automatic system for reducing digestion time [48], but the IMER was not connected to the LC-MS system due to reproducibility considerations. Sproß *et al*. describe a fully automated on-line 3D nano-HPLC/nano-ESI-Q-TOF-MS/MS system composed of a monolithic trypsin reactor in the first dimension, a monolithic affinity column in the second dimension, and C18 HPLC-Chip in the third dimension [49], requiring 4 high performance pumps. In contrast, the OTER-PLOT platform requires only one nanoLC pump with two additional valves that are easily controlled by the MS software.

### Platform demonstration

#### Targeted proteomics

To consider the potential of OTER-PLOT for targeting diagnostic markers, the system must feature high sensitivity and selectivity for the targets in complex samples. In this context, Axin1 and adenomatous polyposis coli (APC) were monitored in biological samples by the OTER-PLOT set-up; Axin1 (96 kDa) and APC (311 kDa) are low abundant, house-keeping proteins of the Wnt-pathway, regulating proteasomal degradation of β-catenin [50], [51]. Several studies have shown that mutations/alterations in either of these two proteins can lead to development of colorectal cancer [52]. Additionally, the levels of both Axin1 and APC have been reported as potential markers for colorectal cancer [53]–[57].

Using high resolution Orbitrap-MS (well suited to selectively target low abundant peptides in the presence of a complex background [58]) with the OTER-PLOT platform, Axin1 was detected in a colon cancer cell (HCT15) lysate within two hours per sample (Figure 6A), with protein identification supported by matching retention time/mass spectra with an external standard. The retention time of the proteotypic Axin1 peptide varied less than 4% between the standard and the complex sample. Combined with the fragmentation mass spectra (Figure S4 A in File S1) the selectivity of this automated assay is high.

Similarly, the platform can be used to detect target proteins in tumor samples, identifying APC in an HCT15 based-tumor (mouse xenograft model) as well as its parent cell line; Figure 6B shows the extracted ion chromatogram (EIC) of a proteotypic peptide from a total protein extract prepared from HCT15 originating tumor tissue and HCT15 cells, using the OTER-PLOT platform. In the present study tumor sample of 500 ng (protein extract) was used for analysis and sufficient to detect APC. Importantly, the OTER-PLOT assay is not susceptible to the mass limitations in WB or antibody selectivity issues, as has been demonstrated for APC.

**Figure 8:**
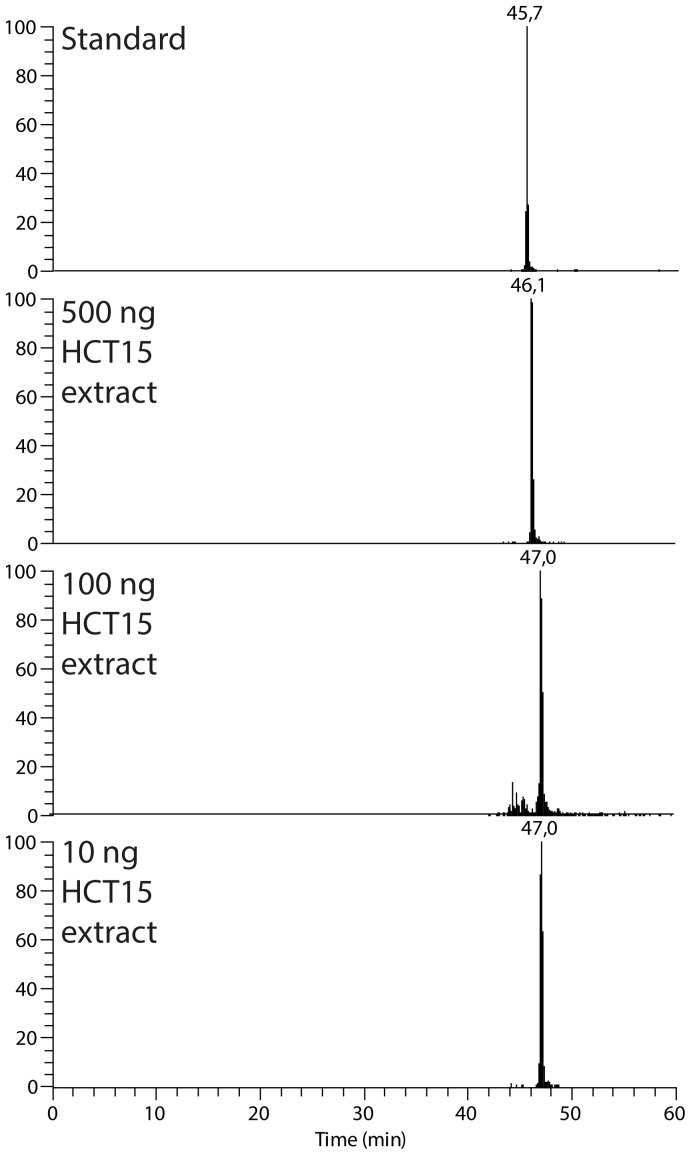
EIC of identified peptide cVDMGcAGLR corresponding to Axin1 in off-line digested standard mixture, 500, 100 and 10 ng HCT15 cell protein extract digested on-line in the nanoproteomic platform. OTER volume was approximately 1.2 µL.

Very narrow open tubular columns should be particularly well suited for handling limited samples; Indeed, the target protein Axin1 could be unambiguously detected (with target peptides easily produced when using OTER) when introducing just 10 ng of sample to the OTER (corresponding to HCT15 cell protein extract from ∼1,000 cells, see experimental section and Methods S3.3 in File S1) (Figure 8), demonstrating the high sensitivity and small sample capability of the platform.

Although the platform was found to have low carry-over, blank injections were routinely performed and evaluated before sample injections.

#### Comprehensive proteomics

**Figure 9:**
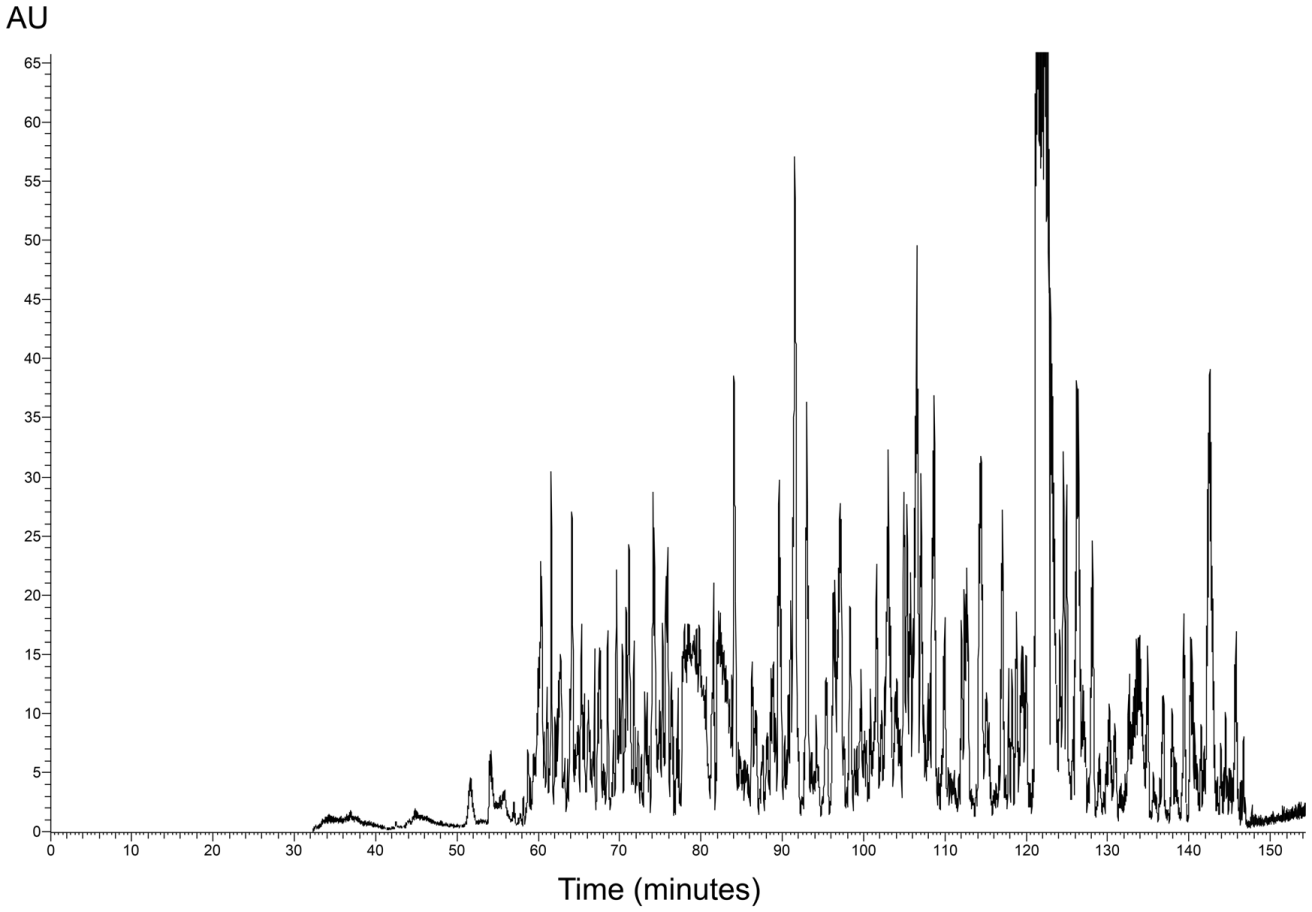
Total ion chromatogram (TIC) of a fully automated digested, separated and detected HCT15 lysate, leading to the identification of ∼1500 proteins (data recorded from gradient start). OTER volume was approximately 1.2 µL.

Even though our primarily goal was to use the OTER-PLOT platform for targeted determinations, it could also be applied for comprehensive analysis. In a single run using a relatively short gradient program (150 min), 1462 proteins were identified in a protein extract (500 ng total protein extract) from HCT15 cells with high peptide confidence, FDR: 0.01 (Figure 9; see also http://www.mn.uio.no/kjemi/supplementary-data/bach/Open-tubular-lab-on-column-mass-spectrometry-for-targe/for raw files and Table S1 for list of identified proteins).

This is a significant improvement compared to similar approaches with integrated IMERs [44] reporting between 78 and 541 proteins identified from much higher amounts of sample digested on-line (e.g. 2.0–2.4 µg [59], [60]); Sun et al reported ∼220 protein IDs when working with similar protein amounts, employing off-line immobilized trypsin digestion [61]. Other central Wnt-pathway proteins were readily identified in the comprehensive mode; such as β-catenin, low density lipoprotein receptor-related protein 6 (LRP5/6), disheveled segment polarity protein 1 (DVL), and casein kinase 2 (CK2), showing that untargeted OTER-PLOT can provide useful complementary information within reasonable time.

## Conclusion

A fully automated open tubular-based nanoproteomic platform, utilizing commercially available instrumentation and equipment, has successfully been developed for sensitive on-line digestion/detection of targeted proteins in biological samples of limited sizes (e.g. ∼1,000 cells). With this workflow, low abundant targets can be monitored in a few hours from sample collection to data analysis. Although, the novel OTER-PLOT platform already performs at a high level, we expect it to have even higher output/selectivity following further optimizations (regarding e.g. column polymer chemistry, column dimensions, affinity column add-ons etc.).

## Supporting Information

File S1 **Chemicals, reagents and materials, methods and supporting figures. Chemicals, reagents and materials S1. Figure S1, Reaction chemistry of the OTER: Polymerization and subsequent immobilization of enzyme through the azlactone functionalities of VDM.** R =  Trypsin/Lys-C. **Figure S2, Sequence coverage (%) of the 10 protein standard mix with digestion at 22°C, 37°C, 50°C and with temperature gradient from 22–50°C in 30 minutes. Figure S3, Sequence coverage (%) as function of digestion time at 50°C (5 min, 15 min, 30 min, 1 h and 2 h). Figure S4, A, Axin1 fragment spectra for peptide TSVQPSHLFIQDPTMPPHPAPNPLTQLEEAR in standard, off-line digest and on-line digest.** B, APC fragment spectra for peptide HETGSHDAER in protein extracts from HCT15 cell line and HCT15 xenograft, respectively. **Figure S5, Manual system used in the development of the platform. Methods S1, Preparation of PLOT and monolithic capillary columns. Methods S2, The nanoproteomic platform, manual system. Methods S3, Standard solutions and sample preparations. Methods S4, Database search.**
(DOCX)

Table S1 **List of identified proteins in comprehensive proteomics experiment.**
(XLSX)

Animation S1 **Operation of automated OTER-PLOT platform.**
(MOV)

Video S1 **Step-by-step production of open tubular enzymatic reactor (OTER).**
(MOV)
