# Supplementary material for: Open Tubular Lab-On-Column/Mass Spectrometry for Targeted Proteomics of Nanogram Sample Amounts
Source: PLoS One. 2014 Sep 15;9(9):e106881. doi: 10.1371/journal.pone.0106881 (PMC4164520; doi:10.1371/journal.pone.0106881)
Supplement: File S1 — Chemicals, reagents and materials, methods and supporting figures. Chemicals, reagents and materials S1. Figure S1, Reaction chemistry of the OTER: Polymerization and subsequent immobilization of enzyme through the azlactone functionalities of VDM. R = Trypsin/Lys-C. Figure S2, Sequence coverage (%) of the 10 protein standard mix with digestion at 22°C, 37°C, 50°C and with temperature gradient from 22–50°C in 30 minutes. Figure S3, Sequence coverage (%) as function of digestion time at 50°C (5 min, 15 min, 30 min, 1 h and 2 h). Figure S4, A, Axin1 fragment spectra for peptide TSVQPSHLFIQDPTMPPHPAPNPLTQLEEAR in standard, off-line digest and on-line digest. B, APC fragment spectra for peptide HETGSHDAER in protein extracts from HCT15 cell line and HCT15 xenograft, respectively. Figure S5, Manual system used in the development of the platform. Methods S1, Preparation of PLOT and monolithic capillary columns. Methods S2, The nanoproteomic platform, manual system. Methods S3, Standard solutions and sample preparations. Methods S4, Database search. (DOCX) [file pone.0106881.s001.docx]

File S1

Open tubular lab-on-column/mass spectrometry for targeted proteomics of nanogram sample amounts

Hanne Kolsrud Hustoft^1^, Tore Vehus^1*^, Ole Kristian Brandtzaeg^1^, Stefan Krauss^2^, Tyge Greibrokk^1^, Steven Ray Wilson^1^ and Elsa Lundanes^1^

^1^Department of Chemistry, University of Oslo, Oslo, Norway

^2^Unit for Cell Signaling, Cancer Stem Cell Innovation Center, Oslo University Hospital, Oslo, Norway

* Corresponding author e-mail: toreveh@mn.uio.no

# Chemicals, reagents and materials

**Chemicals, reagents and materials** **S1:**

HPLC-grade acetonitrile (ACN, VWR (West Chester, PA, USA)), HPLC water Chromasolv plus for HPLC, Sigma Aldrich (St. Louis, MO, USA), formic acid (FA, 50 %, Fluka, by Sigma Aldrich), ammonium acetate (98 %, Sigma Aldrich) and ammonia (28 %, VWR Fontenay-sous-Bois, France) were used to prepare the mobile phases. All proteins were reduced and alkylated prior to on-line digestion in the proteomic platform with triethylammonium bicarbonate buffer (pH: 8.5±0.1) (tABC, Sigma-Aldrich), DL-dithothreitol, (DTT, Fluka, Sigma Aldrich), iodoacetamide (IAM, Sigma Aldrich). Sodium phosphate monobasic (99 %, Sigma Aldrich) was used to prepare the phosphate buffer. The standard proteins used were cytochrome C (Cyt C) (Bos Taurus, 11.7 kDa), hemoglobin (Hemo) (Homo Sapiens, 15.2 kDa), myoglobin (Myo) (Equine heart, 17 kDa), carbonic anhydrase (CaA) (Bos Taurus, 29.1 kDa) immunoglobulin G (IgG) (Homo sapiens, 36.1 kDa), serum albumin (HSA) (Homo Sapiens, 69.3 kDa), transferrin (Transf) (Homo sapiens, 77 kDa) and fibrinogen (Fibr) (Bos Taurus, 340 kDa). The proteins were all obtained from Sigma-Aldrich. Beta-catenin (β-cath) (Homo sapiens, 85.4 kDa) was obtained from Millipore Corporation (Billercia, MA, USA). Recombinant ProGRP isoform 1 was obtained as described by Torsetnes *et al.*[[1](#_ENREF_1)]. Recombinant Axin1 (H00008312-Q02) and APC (H00000324-Q01) were obtained from Abnova (Tapei City, Taiwan). For preparation of the columns the following were used: N,N-dimethylformamide anhydrous (DMF), 3-(trimethoxysilyl)propyl methacrylate (γ-MAPS, 98 %), divinylbenzene (DVB, 80 % mixture of isomers, styrene, 99 %), 1-dodecanol, 1-decanol and 1-heptanol, (98 %), sodium hydroxide (NaOH, 99 %), inhibitor 2,2-diphenyl-1-picrythydrazyl hydrate (DPPH), ethylene dimethacrylate (EDMA, 98 %), 2-hydroxyethyl methacrylate (HEMA, 97 %, containing 200-220 ppm monomethyl ether hydroquinone as inhibitor) and initiator 2,2´ azobis(2-methylpropinonitrile) (AIBN), all purchased from Sigma Aldrich. Vinyl azlactone (VDM) was purchased from Polysciences Inc. (Warrington, PA, USA). Toluene was purchased from Rathburn Chemical Ltd. (Walkerburn, Scotland, UK, while ethanol was purchased from Arcus (Oslo, Norway). Trypsin from bovine pancreas (≥ 10,000 BAEE), benzamidine (>95 %), and ethanolamine (99 %) were all purchased from Sigma Aldrich. Lys-C and Trypsin/Lys-C mix were purchased from Promega Corporation (Madison, WI, USA). Nitrogen gas (99.99 %) was obtained from AGA (Oslo, Norway).

HCT15 cells were from ATCC (LGC Standards, Queens Road, Middlesex, UK). RPMI1640, fetal bovine serum (FBS), penicillin-streptomycin and trypsin-EDTA were acquired from Invitrogen (Life Technologies, Carlsbad, CA, USA). 1X phosphate buffered saline solution, MgCl_2_, Tris-HCl pH 8 and NaCl solutions were made by Oslo University Hospital (Oslo, Norway). Protease inhibitor cocktail tablets were from Roche Diagnostics (Basel, Switzerland) and urea trifluoroacetic acid (TFA) was from Sigma Aldrich. The C4 solid phase extraction cartridges were from Perkin Elmer (Waltham, MA, USA) while the Bond Elute C18 SPE cartridges were from Agilent (Santa Clara, CA, USA). Polyimide coated fused silica tubing (360 μm outer diameter (OD), 100, 75, 50, 20, 15 and 10 μm ID) were purchased from Polymicro Technologies (Phoenix, AZ, USA).

# **Methods**

## **Methods S1: Preparation of PLOT and monolithic capillary columns**

All columns were made in pre-treated and silanized capillaries (Video: *OTERpreparation.mov*).

### Methods S1.1: Pre-columns

#### Methods S1.1.1: PS-DVB

50 μm ID monolithic pre-columns were prepared by polymerization of 21 % (wt.%) styrene with 19 % (wt.%) DVB and 1% (with respect to monomer) initiator AIBN. The binary porogenic mixture of 13 % toluene and 47 % 1-dodecanol (wt.%) was based on a preparation described by Lv *et al*.[[2](#_ENREF_2)] The polymerization temperature was 70°C with a duration time of 20 h.

#### Methods S1.1.2: BuMA

The polymerization mixture consisted of 0.24 g BuMA, 0.3400 g 1-propanol, 0.26 g 1,4-butanediol, 0.16 g EDMA and 0.004 g AIBN. The polymerization mixture was sonicated for 5 minutes before filled into 50 μm ID capillary. The capillary was polymerized in a GC oven at 70 ^°^C for 16 h. The column was flushed with ACN for 30 min and dried with N_2_ for 1 h.

## Methods S1.2: PLOT column

The PLOT columns, with polymeric layer ~0.75 µm were prepared as described by Rogeberg *et al.* [[3](#_ENREF_3)] The column ID was 10 μm and the column length was 5 meter.

## Methods S2: The nanoproteomic platform, manual system

Three pumps were used in the platform (Figure S5). Pump 1, an Agilent 1100 isocratic pump (Agilent, Sao Paulo, CA), was used for introducing the sample into the OTER with a 0.5 μL/min flow for 1 min (or adjusted to the volume of the OTER (~60-300 nL)). The sample (added 50 mM tABC buffer and 5 % ACN) was digested for 30 min at 37°C. The blank used between the runs had the same buffer and ACN composition in order to monitor possible carry-over. For trapping of peptides generated in the OTER onto the pre-column, a 1200 series Agilent pump (pump 2) with an Agilent G1379A series degasser was used. The flow was 0.5 µL/min, and peptides were trapped for 4 min. For gradient elution, an 1100 series Agilent pump (Pump 3) with a G1379A series degasser was used and the flow set to 2 μL/min. A 10-port VICI valve (Valco Instruments, Houston, TX) and two 6-port valves (valve 2 and 3) (Valco) were used in the platform. The 10-port valve was placed inside a column oven (Mistral, Spark Holland) at 37°C. Splitting of the flow from pump 3 was achieved by valve 2 with a ratio of 1:50, resulting in a PLOT column flow rate of 40 nL/min. The PLOT column was connected to a PicoTip nanospray tip (with 5 μm ID) with a Picoclear union (both purchased from New Objective (Woburn, MA, USA)). For pump 1 and 2, mobile phase A was comprised of 50 mM NH_4_OAc (pH 8.75) and mobile phase B comprised of ACN/ NH_4_OAc (pH 8.75) (90 %/10 % (v/v)). 4 % B was used for the trapping of peptides onto the pre-column. For the gradient elution (pump 3) mobile phase A was comprised of H_2_O/FA (100 %/0.1 % (v/v)) and mobile phase B comprised of ACN/H_2_O/FA (90 %/10 %/0.1 % (v/v/v)), and gradient elution with 5-40 % B in 20 min and 40 % B from 20 to 28 min was performed.

## **Methods S3: Standard solutions and sample preparations**

### **Methods S3.1: Preparation of standard solutions**

The 10 proteins from human, bovine and equine ranging from ~12 – 340 kDa constituting the standard mixture were dissolved in water, to a total concentration of 300 μg/mL (with 430 μmole of each protein). Reduction was carried out by the addition of DTT, to a final concentration of 5 mM, and incubated at 56 °C for 45 min. The mixture was cooled to room temperature (25 °C) prior to the alkylation. For alkylation, IAM was added to a final concentration of 15 mM, and the sample was incubated at room temperature in the dark for 30 min. Prior to in-solution digestion the sample buffer was adjusted to pH 8.5 by the addition of tABC buffer and 5 % ACN was added.

### Methods S3.2: Preparation of APC and AXIN1 standard peptides

Recombinant Axin1 and APC were each dissolved in 8M urea containing 50 mM Tris-HCl pH 8, and were reduced, alkylated as described above (S3.1), desalted using C18 cartridges (S3.3) and dissolved in 0.1 % TFA in 5 % ACN to a final concentration of 1 ng/µL. The selection of proteotypic peptides were done through analysis of the digested APC/Axin1 protein mixture with the Skyline software (v1.3, MacCoss, University of Washington, Seattle, WA, USA), excluding peptides shorter than 6 amino acids, containing either cysteine or methionine. The suggested peptides were also searched against the human proteome in Uniprot (The Uniprot Consortium).

### Methods S3.3: Preparation of HCT15 cell lysate for on- and off-line digestion

The HCT15 cells were cultured in RPMI1640 media supplemented with 10 % and 60U/mL penicillin-streptomycin and harvested with trypsin-EDTA at 80 percent confluency. The cells were washed 3 times in phosphate-buffered saline solution prior to protein extraction. Proteins were lysed in ice-cold lysis buffer containing 8 M urea, 50 mM Tris-HCl pH 8, 2 mM MgCl_2_, 40 mM NaCl and 1X protease inhibitor cocktail. The cell pellet was homogenized and incubated on ice for 30 minutes and sonicated briefly before cell debris were removed by centrifugation at 13000 g for 15 minutes at 4°C. The supernantant was removed and proteins reduced with 5 mM DTT (final concentration) at 37°C for 60 minutes and alkylated with 15 mM IAM (final concentration) for 30 minutes in the dark. The sample was diluted with 50 mM tABC buffer and 5 % ACN to protein concentrations from 10 to 500 ng/µL. 10 μL of each sample were transferred to autosampler vials. Before on-line digestion the samples were desalted using C4 solid phase extraction (SPE) cartridges. Briefly, the cartridges was activated with 1 mL ACN containing 0.1 % (v/v) TFA and equilibrated with 1 mL 0.1 % TFA/ACN (98/2, v/v). The sample was added to the cartridge which was washed with 1 mL 0.1 % TFA/ACN (98/2, v/v) before eluted with 0.1 % (v/v) TFA in ACN. The sample was dried using SpeedVac (Thermo Fisher Scientific, former Savant) and reconstituted in 4 % ACN in 50 mM tABC. Working samples with concentrations from 0.5 – 1 mg/mL were prepared.

Off-line digestion was performed for 1 hour using 1:20 protein enzyme ratio of Trypsin/Lys-C in 8-fold diluted lysis buffer diluted with 50 mM Tris-HCl pH 8 after reduction and alkylation as described above. The peptides were desalted using C18 SPE cartridges with the same procedure as described above. The samples were evaporated to dryness and reconstituted to working concentrations of 0.5-1 mg/mL in 0.1 % TFA/ACN (98/2, v/v).

### Methods S3.4: Preparation of tumor tissue samples

The tumor tissue was acquired from Dr. Waaler (Oslo University Hospital) and it was homogenized in 6 times the mass of the sample with ice cold lysis buffer in a MagNaLyzer instrument (Roche) at 4000 rpm 4 times for 15 seconds. The sample was then centrifuged at 13 000 g for 15 minutes at 4°C, incubated for 15 minutes at 4°C and re-centrifuged. The supernatant was transferred to new Eppendorf tubes and proteins reduced, alkylated, desalted and reconstituted as described above (S3.4).

All animal experiments have been approved by the Norwegian Animal Research Authority (NARA) and registered by the Authority.

### Methods S3.5: Determination of protein and peptide concentrations

The protein concentrations were determined at 280 nm with bovine serum albumin (BSA) as standard with a NanoDrop instrument (Thermo Scientific). The peptide concentrations in the digested samples were determined using absorbance at 205 nm with settings as defined elsewhere [[4](#_ENREF_4)].

## Methods S4: Database search

Thermo Proteome Discoverer (v1.4) was used for database search with Sequest against the Uniprot human database and Mascot against the SwissProt human database (Uniprot Consortium). The search was either performed with Lys-C or trypsin as cleavage reagent with maximum two missed cleavages allowed. The search results were then merged. The precursor mass tolerance was set to 10 ppm and fragment mass tolerance to 0.8 Da. Methionine oxidation and N-terminal acetylation were set as dynamic modifications, whereas carbamiodomethylation of cysteine was set as a fixed modification. A false discovery rate (FDR) of 1 % was used to filter the results.

For identification of target proteins in the APC/Axin1 standard mixture and the targeted LC-MS/MS analysis, the Sequest algorithm was applied as described above, using the sequences obtained from Uniprot database, P25054 for APC and O15169 for Axin1.

# Figures





Figure S1: Reaction chemistry of the OTER: Polymerization and subsequent immobilization of enzyme through the azlactone functionalities of VDM. R= Trypsin/Lys-C.


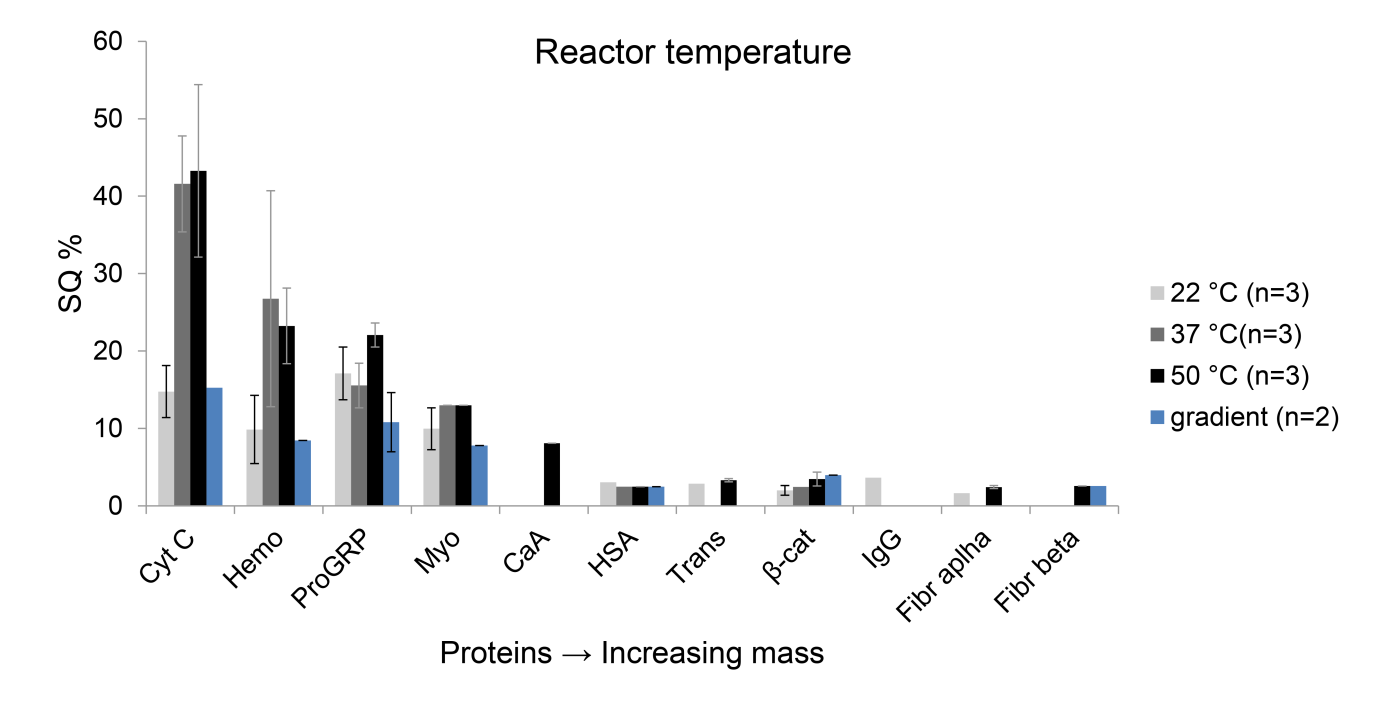


Figure S2: Sequence coverage (%) of the 10 protein standard mix with digestion at 22 °C, 37 °C, 50 °C and with temperature gradient from 22-50 °C in 30 minutes.


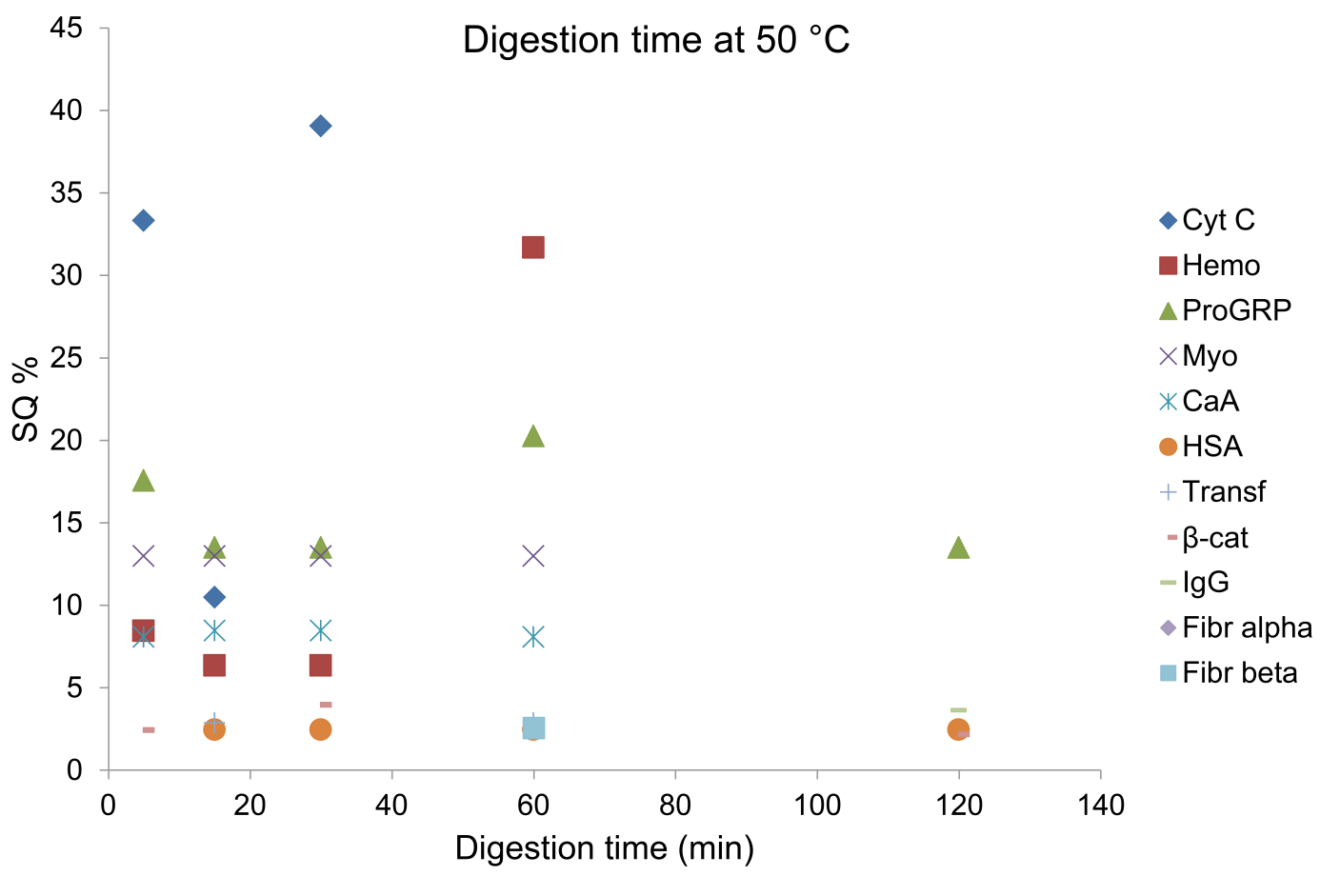


Figure S3: Sequence coverage (%) as function of digestion time at 50 °C (5 min, 15 min, 30 min, 1 h and 2 h).


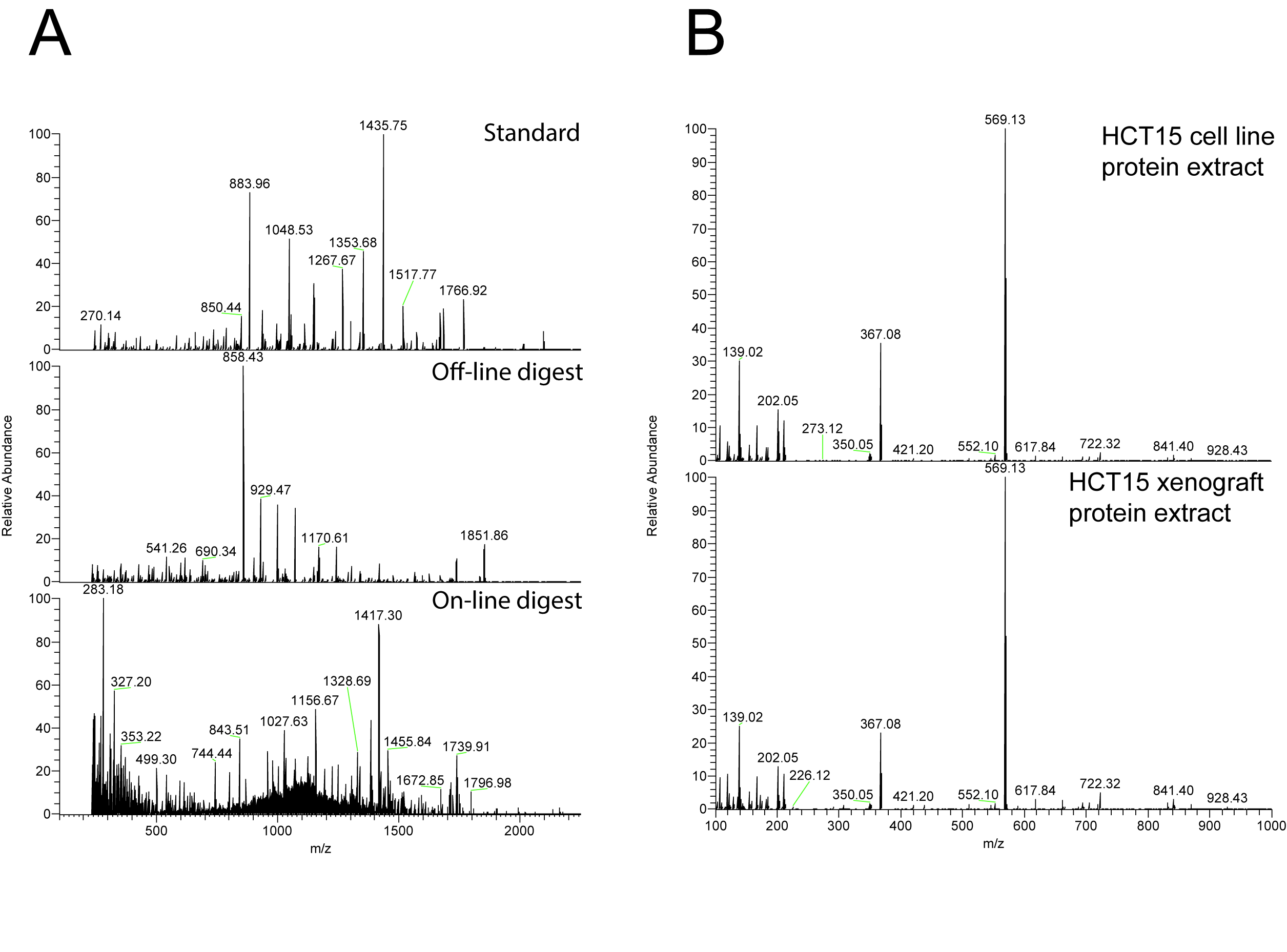


Figure S4: A, Axin1 fragment spectra for peptide TSVQPSHLFIQDPTMPPHPAPNPLTQLEEAR in standard, off-line digest and on-line digest. B, APC fragment spectra for peptide HETGSHDAER in protein extracts from HCT15 cell line and HCT15 xenograft, respectively.


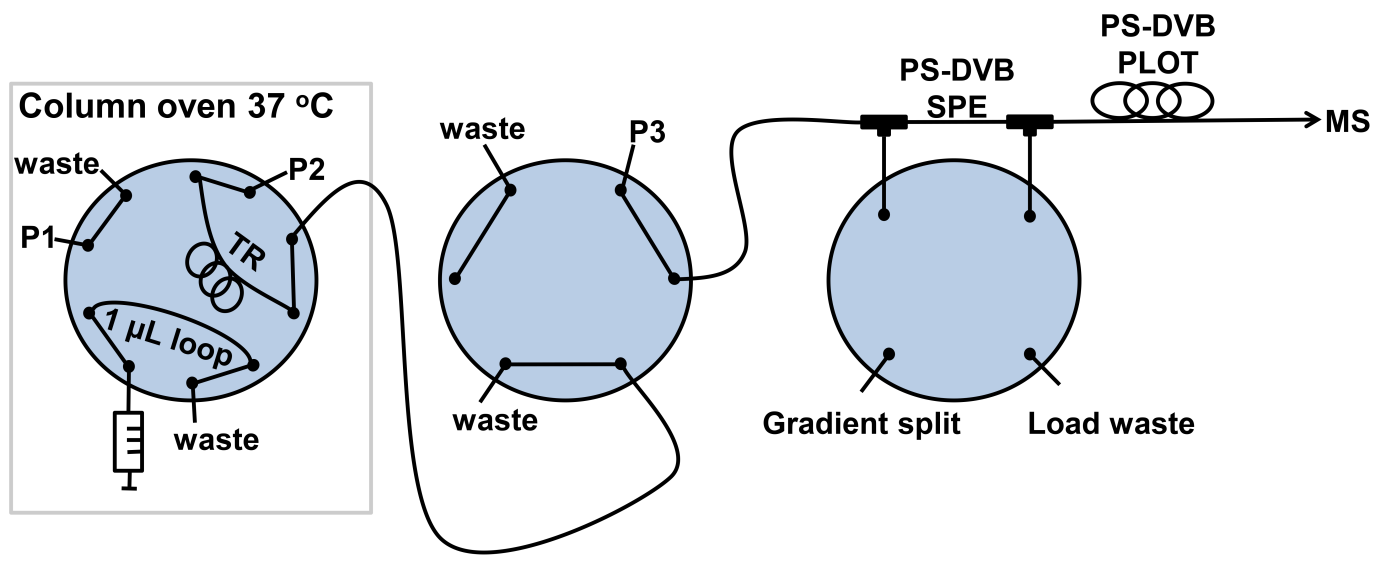
Figure S5: Manual system used in the development of the platform.

1. Torsetnes SB, Nordlund MS, Paus E, Halvorsen TG, Reubsaet L (2013) Digging deeper into the field of the small cell lung cancer tumor marker ProGRP: a method for differentiation of its isoforms. J Proteome Res 12: 412-420.

2. Lv Y, Lin Z, Svec F (2012) Hypercrosslinked large surface area porous polymer monoliths for hydrophilic interaction liquid chromatography of small molecules featuring zwitterionic functionalities attached to gold nanoparticles held in layered structure. Anal Chem 84: 8457-8460.

3. Rogeberg M, Wilson SR, Greibrokk T, Lundanes E (2010) Separation of intact proteins on porous layer open tubular (PLOT) columns. J Chromatogr A 1217: 2782-2786.

4. Scopes RK (1974) Measurement of protein by spectrophotometry at 205 nm. Anal Biochem 59: 277-282.
